# Maladaptive neurovisceral interactions in patients with Internet gaming disorder: A study of heart rate variability and functional neural connectivity using the graph theory approach

**DOI:** 10.1111/adb.12805

**Published:** 2019-07-12

**Authors:** Su Mi Park, Ji Yoon Lee, A Ruem Choi, Bo Mi Kim, Sun Ju Chung, Minkyung Park, In Young Kim, Jinsick Park, Jeongbong Choi, Sung Jun Hong, Jung‐Seok Choi

**Affiliations:** ^1^ Department of Psychiatry SMG‐SNU Boramae Medical Center Seoul South Korea; ^2^ Department of Clinical Medical Sciences Seoul National University College of Medicine Seoul South Korea; ^3^ Department of Psychiatry and Behavioral Science Seoul National University College of Medicine Seoul South Korea; ^4^ Department of Biomedical Engineering Hanyang University Seoul South Korea; ^5^ Medical Device Development Center Osong Medical Innovation Foundation Cheongju South Korea

**Keywords:** autonomic nervous system, functional connectivity, heart rate variability, Internet gaming disorder, neurovisceral integration

## Abstract

Heart rate variability (HRV) can be used to represent the regulatory adaptive system and is a proxy for neurovisceral integration. Consistent with the view that, like other addictions, Internet gaming disorder (IGD) involves disrupted regulatory function, the present study hypothesized that IGD patients would show (a) decreased HRV, (b) ineffective functional neural connectivity, and (c) differential patterns of association between HRV and functional neural connectivity relative to healthy controls (HCs). The present study included 111 young adults (53 IGD patients and 58 age‐ and sex‐matched HCs) who underwent simultaneous recordings with an electrocardiogram and electroencephalogram during a resting state. Heart rate (HR), HRV, and functional neural connectivity were calculated using the graph theory approach. Compared with the HCs, the IGD patients exhibited elevated HR and decreased HRV based on the high frequency (HF), which reflects suppression of parasympathetic and/or vagal tone. The IGD patients also exhibited a heightened theta band characteristic path length (CPL) compared with HCs, indicating decreased efficacy of the functional network. Furthermore, IGD patients exhibited negative correlations between the standard deviation of the normal‐to‐normal interval index (SDNNi) and theta and delta CPL values, which were not observed in HCs. In conclusion, the present findings suggest that IGD patients might have maladaptive brain‐body integration features involving disruptions of the autonomic nervous system and brain function.

## Graphical abstract

Compared to healthy controls (HCs), Internetgaming disorder (IGD) patients showed decreased heart ratevariability (HRV) as represented by the high frequency (HF) and heightenedtheta band characteristic path length (CPL), indicating decreased efficacyof the functional network. IGD patients exhibited negative correlationsbetween the standard deviation of the normal‐to‐normal interval index(SDNNi) and theta and delta CPL. The findings suggest that IGD patients havemaladaptive brain–body integration features involving disruptions ofthe autonomic nervous system and brain function.

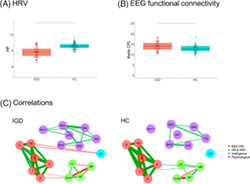

## 1 INTRODUCTION

Because of the development of modern information technology, the Internet has become an integral part of our lives. Although the convenience of everyday life has increased, the adverse effects of overusing the Internet have also become a social issue. In particular, compulsive playing of Internet games is now regarded as an addiction‐related mental disorder that leads to physiological, psychological, and social adjustment problems such as substance use disorder (SUD), gambling disorder, and symptoms of cravings and withdrawal.^1^ Consistent with this notion, the Diagnostic and Statistical Manual of Mental Disorders‐Fifth Edition (DSM‐5)^2^ proposed that Internet gaming disorder (IGD) is a psychiatric disorder that will require further research. Additionally, the 11th revision of the International Classification of Diseases (ICD‐11), which was released in 2018, includes a definition for gaming disorder.^3^

Heart rate variability (HRV) is an index of the autonomic nervous system (ANS) that represents the activation of sympathetic and parasympathetic nerves, including the vagus nerve. Reduced HRV is predictive of cardiovascular disease and mortality^4^ and is commonly regarded as indicative of decreased efficiency of autonomic control related to stress.^5^ Additionally, according to the model of neurovisceral integration, HRV may provide a window into human emotional regulation or dysregulation through interactions with the brain.^6^ To regulate behavior and emotion, the human body and brain receive signals from external stimuli and then interact with each other through a feedback loop that includes the following two paths: (a) the afferent stream, which refers to input from the body to the brain, and (b) the efferent stream, which refers to output from the brain to the body. Neuroimaging studies have confirmed an association between HRV and neural structures in the central autonomic network (CAN), including connections between prefrontal cortical regions and the amygdala.^7^, ^8^

The following parameters are recommended as the standard and best‐characterized HRV metrics in terms of clinical fields: high‐frequency (HF) and low‐frequency (LF) power calculated using power spectral density analysis and the standard deviation of the normal‐to‐normal beat interval (SDNN).^9^ The HF is considered a representative index of HRV in that efferent vagal activity is a key factor in HF.^10^ LF power is generally assumed to work with the sympathetic‐parasympathetic nervous system, with a dominant sympathetic component. The SDNN is the time domain index of HRV, reflecting a flexible cardiac response to adapt to external changes. It is widely accepted that decreased HF and a reduction of SDNN are representative of dysfunctional HRV, indicating that the organism is under pressure or in a stressful condition. Hence, decreased HRV is often found in psychiatric disorders.^11^

Addictive processes, including dysfunction within regulatory systems, can be approached from the perspective of brain‐body integration. For example, the HRV of SUD patients can be affected by changes in neurovisceral dynamics, arousal modulation, baroreflex sensitivity, and stress reactivity, which can ultimately lead to compulsive addictive behaviors, similar to being directly affected by substance use or intoxication.^12^ A decreased HRV in individuals with an SUD, including alcohol and cigarette disorders, reflects dysfunctional parasympathetic and vagal tone.^13^, ^14^ One study did not find differences in HRV between alcoholic and control subjects but suggested that HRV accounts for the degree of cravings for substances, including alcohol, even after controlling for the amount of alcohol assumption.^15^ Similar to SUD studies, other recent studies have observed decreased HRV in IGD patients and heavy Internet users that are represented as HF or the SDNN.^16^, ^17^

Along with the concept that “our brain is a network,”^18^ recent neurobiological studies in the psychiatric field have shed light on functional connectivity, which is defined as the co‐occurring temporal dynamics of neural activity patterns in separate brain regions.^19^ In addition, in recent decades, large‐scale resting‐state functional connectivity has rapidly become a central theme, as it reflects holistic functional characteristics and is associated with a behavioral phenotype and trajectories of psychiatric disorders.^18^, ^20^, ^21^ Addiction is closely related to abnormal functional connectivity; dispositional vulnerability of the brain may lead to a behavioral addiction (eg, compulsive pleasure seeking), and addictive behavior may cause changes in the brain.^22^ In particular, previous studies have indicated that heterogeneous drug‐use cohorts exhibit disturbed reward circuity (eg, in amygdala‐ventromedial prefrontal cortex [VMPFC] regions). Emotional and cognitive dysregulation can also accompany decreased reward circuity and suppress the default mode network (eg, posterior cingulate cortex [PCC] and anterior cingulate cortex [ACC]), activating a “task‐negative” condition, which involves disrupting integrated cognitive and emotional processing, mind wandering, and self‐referential processing.^23^

In cases of IGD and SUD, a resting‐state functional magnetic resonance imaging (fMRI) study that applied graph theory to IGD patients showed that the functional connectivity features of these patients are close to a pathological state.^24^ Yuan et al^25^ observed reductions in resting‐state frontostriatal functional connectivity in IGD patients, which is correlated with cognitive control deficits. Similarly, Ko et al^26^ demonstrated that functional connectivity between the amygdala and dorsolateral prefrontal cortex (DLPFC) could indicate vulnerability to the features of IGD, such as impulsivity. Taken together, these findings suggest that IGD is associated with dysfunctional brain networks, which is a notion that many studies have demonstrated.

As noted earlier, HRV can be considered a window into the brain‐body connection. However, addiction studies investigating neurovisceral dynamics via neuronal HRV correlates are rare, whereas studies using brain imaging modalities or HRV have been widely conducted.^22^, ^27^ Electroencephalography (EEG), a non‐invasive index of neurodynamics with high time resolution, has advantages because it can be recorded simultaneously with HRV data (EEG‐HRV). Several psychiatric studies have observed correlates of the HRV‐EEG relationship. A study assessing posttraumatic stress disorder (PTSD) found that decreases in the high HF HRV of parasympathetic nerves and increases in the EEG alpha spectrum were features of PTSD^28^; however, those authors did not describe correlates of HRV‐EEG interaction. Another recent study that performed a pathway analysis of nonclinical participants suggested that childhood trauma affects emotional lability by increasing LF HRV via the EEG beta spectrum.^29^

Our research team previously reported that patients with an Internet addiction showed high impulsivity and aggressiveness and found that depression, anxiety, and impulsivity affected Internet addiction severity, along with behavioral motivation and interpersonal difficulty.^30^, ^31^, ^32^, ^33^ In addition, many previous empirical studies have suggested that these variables related to psychological well‐being are related to cardiovascular and neuronal functional activities (eg, Beauchaine,^34^ Fingelkurts et al,^35^ and Hofman and Schutter^36^). Intellectual performance is also known to be associated with the global efficiency of brain functional connectivity, pointing to the merits of considering these domains together when looking at functional connectivity.^37^

The present study clarified the nature of the dysfunctional pattern of neurovisceral connections in IGD patients compared with healthy controls (HCs) using simultaneous measurements of HRV and EEG data in a resting‐state condition. To measure EEG functional connectivity, the graph theory approach was adopted for large‐scale global integration of the network. The following three hypotheses were tested: (a) IGD patients would show decreased HRV (HF and SDNN) compared with HCs, (b) IGD patients would show decreased efficiency in functional neural connectivity, and (c) the relationships of HRV‐EEG functional neural connectivity patterns in IGD patients would be different from that in HCs in that decreased HRV would be positively associated with disrupted functional connectivity. Additionally, psychological variables associated with IGD (ie, impulsivity, depression, anxiety, aggression, and behavioral motivation) and global intelligence were assessed to explore the relationships among these variables.

## 2 METHODS AND MATERIALS

### 2.1 Participants

The present study included 55 patients (52 males and three females) diagnosed with IGD based on DSM‐5 criteria who were recruited from the outpatient clinic of SMG‐SNU Boramae Medical Center; all IGD patients were medication naïve. Additionally, 60 age‐ and sex‐matched HC participants (55 males and five females) with no history of psychiatric illness who played Internet games for less than 2 hours per day were recruited from the local community. The Structured Clinical Interview for the DSM‐IV (SCID‐IV) was administered to identify past and present psychiatric illnesses. The exclusion criteria consisted of neurological disease, significant head injury accompanied by loss of consciousness, medical illness with documented cognitive sequelae, sensory impairment, cardiovascular disease, and/or intellectual disability (intelligence quotient [IQ] less than 70). Patients with a past or present history of a primary diagnosis of a major psychiatric disease, including major depressive disorder, bipolar spectrum disorder, or psychotic disorder, were also excluded from the study. Additionally, two IGD patients were excluded because their IQ was less than 70, and two HC participants were excluded because their electrocardiogram (ECG) results indicated suspected arrhythmia. Thus, 53 IGD patients (50 males and three females; mean age: 23.55 ± 4.88 years) and 58 HCs (53 males and five females; mean age: 24.90 ± 3.40 years) were included in the final analyses.

Table 1 shows the demographic characteristics of the IGD and HC groups. All participants completed informed consent forms prior to participation. The study was conducted in accordance with the Declaration of Helsinki, and all study protocols were approved by the Institutional Review Board of no. 16‐2014‐139. Data were collected from October 2014 to September 2018.

**Table 1:** Group differences in demographic and psychological variables

	IGD (n = 53)		HC (n = 58)		*t*	*P*	*η*_*p*_^*2*^
	Mean	SD	Mean	SD			
Age	23.55	4.88	24.90	3.40	−1.70	.091	‐
Sex, n	M = 50, F = 3		M = 53, F = 5		‐	‐	‐
Education	12.88	1.56	14.63	1.82	−5.34	<.001	0.21
IQ	104.78	17.68	117.86	10.96	−4.68	<.001	0.17
Time_D	8.59	7.41	0.78	1.11	9.64	<.001	0.37
Time_W	10.02	7.6	1.28	2.31	11.81	<.001	0.39
IAT	64.63	16.72	29.98	8.26	13.46	<.001	0.64
BDI2	18.75	11.85	3.53	3.91	7.54	<.001	0.44
BAI	16.35	13.97	4.51	4.58	4.76	<.001	0.26
AQ	74.59	16.91	54.25	11.77	6.27	<.001	0.34
BIS11	67.18	10.61	55.51	7.61	6.04	<.001	0.29
BIS	21.63	4.60	17.56	3.89	4.58	<.001	0.19
BAS	35.02	6.79	32.77	6.54	1.19	.235	‐
AUDIT	5.09	4.64	5.05	3.38	0.05	.964	‐

Abbreviations: AQ, Korean version of the Buss‐Perry Aggression Questionnaire; AUDIT, Korean version of the Alcohol Use Disorder Identification Test; BAI, Korean version of the Beck Anxiety Inventory; BDI2, Korean version of the Beck Depression Inventory‐2; BIS/BAS, Korean version of the Behavioral Inhibition System/Behavioral Approach System (BIS/BAS) scales; BIS11, Korean version of the Barrett Impulsiveness Scale‐11; HC, healthy control; IAT, Korean version of the Young Internet Addiction Test; IGD, Internet gaming disorder; IQ, intelligence quotient; Time_D, time spent for Internet gaming during weekday per hour/day; Time_W, time spent for Internet gaming during weekend per hour/day.

### 2.2 Assessments

#### 2.2.1 Self‐reported psychological data

##### Korean version of the Young Internet Addiction Test

The Young Internet Addiction Test (Y‐IAT), which was developed by Young^38^ and translated to Korean by Lee et al,^39^ is a 20‐item self‐report questionnaire. Each item is scored using a 5‐point scale ranging from 1 (*very rarely*) to 5 (*very frequently*) such that a higher score reflects a greater tendency toward IGD symptoms. The Cronbach alpha value in this study was .97.

##### Korean version of the Beck Depression Inventory‐2

The Beck Depression Inventory‐2 (BDI2), which was developed by Beck et al^40^ and translated to Korean by Kim et al,^41^ is a 21‐item self‐report questionnaire that measures the severity of particular symptoms experienced over the past week. Total scores range from 0 to 63 such that a higher score reflects more severe depression. The Cronbach alpha value in this study was .95.

##### Korean version of the Beck Anxiety Inventory

The Beck Anxiety Inventory (BAI), which was developed by Beck et al^42^ and translated to Korean by Kim et al,^43^ is a 21‐item self‐report questionnaire that evaluates anxiety. Total scores range from 0 to 63 such that a higher score reflects more severe anxiety. The Cronbach alpha value in this study was .95.

##### Korean version of the Buss‐Perry Aggression Questionnaire

The Aggression Questionnaire (AQ), which was developed by Buss and Perry^44^, ^45^ and translated to Korean by Seo & Kwon,^46^ is a 29‐item instrument on which participants rate statements along a 5‐point continuum from 1 to 5 such that a higher score reflects more severe aggressiveness. The Cronbach alpha value in this study was .91.

##### Korean version of the Barratt Impulsiveness Scale‐11

The Barratt Impulsiveness Scale‐11 (BIS11) is a revised version of the original Barratt Impulsiveness Scale that includes 11 items used to assess the degree of impulsivity^47^, ^48^ and translated to Korean by Lee et al.^49^ The BIS11 items are scored on a 4‐point Likert scale (1 to 4) such that a higher score is indicative of high impulsiveness. The Cronbach alpha value in this study was .84.

##### Korean version of the Behavioral Inhibition System/Behavioral Approach System scale

The Behavioral Inhibition System/Behavioral Approach System (BIS/BAS) scale is used to assess sensitivity to rewards and punishments^50^ and translated to Korean by Kim & Kim.^51^ This measure consists of 20 items that are rated on a 4‐point Likert scale from *totally agree* to *totally disagree*; the BIS scale consists of seven items, and the BAS scale consists of 13 items. The Cronbach alpha value for BIS was .88 and for BAS was .82.

##### Korean version of the Alcohol Use Disorder Identification Test

The Alcohol Use Disorder Identification Test (AUDIT) was used to assess the comorbidity and severity of alcohol use disorder (AUD)^52^ and translated to Korean by Kim et al.^53^ This scale measures the frequency of alcohol abuse behavior and contains 10 questions, scored on a 4‐point Likert scale. The Cronbach alpha value in this study was .78.

#### 2.2.2 Intelligence

##### Korean version of the Wechsler Adult Intelligence Scale—Fourth Edition (K‐WAIS–IV)

In the present study, cognitive ability and IQ were assessed with the Korean version of the Wechsler Adult Intelligence Scale—Fourth Edition (K‐WAIS‐IV).^54^, ^55^ Because the first participants to be enrolled (19 IGD patients and 21 HCs) were assessed using an earlier version of the K‐WAIS,^56^ linear regression analyses of the IQ score of each group were conducted separately to evaluate differences based on WAIS version; there were no significant differences (*P* > .05).

#### 2.2.3 Heart rate and HRV

##### ECG data collection

Inter‐beat intervals (IBI) during a 5‐minute period were obtained during EEG recording while the participants were in a relaxed and seated position with their eyes closed. The sampling rate of the recording was 1000 Hz. ECG signals were recorded using two sintered Ag/AgCl electrodes placed on the left and right supraclavicular areas. The participants were required to restrict their consumption of caffeine and nicotine for 2 hours prior to the experiment.

##### Heart rate and HRV parameters

All R‐R intervals were automatically extracted using the Pan and Tomkins algorithm.^57^ Prior to the HRV frequency analysis, the R‐R interval data were preprocessed to remove the ectopic beat using the 20% filter and then interpolated with 4 Hz. Then, HRV was transformed into a power spectrum using an autoregressive spectral analysis, and the following parameters were extracted from the measured ECG signals with HRV analysis software (HRVAS; https://sourceforge.net/projects/hrvas/) in the MATLAB environment: (a) heart rate (HR), which was the number of heart contractions per minute (bpm); (b) frequency domain HRV, which was defined as LF (0.04 to 0.15 Hz) and HF (0.15 to 0.4 Hz); and (c) time domain HRV, which was defined as the SDNN (normal R‐R), the average of the SDNN for each 50‐second segment (SDNN index [SDNNi]) reflecting short‐term HRV variability, the root mean square of the difference of the NN intervals (RMSSD), and the percentage of NN intervals that differed by more than 50 milliseconds (pNN50). The SDNN is the simplest variable to calculate the time domain HRV, and the length of the analyzed recording may affect the SDNN.^58^ Thus, in addition to SDNN, the above time domain indices, which are often used in the clinical field, were selected,^59^ according to a recommendation from the Task Force of the European Society of Cardiology and the North American Society of Pacing and Electrophysiology.^10^

#### 2.2.4 EEG network

##### EEG data collection

Resting‐state EEG data were recorded for 5 minutes in an electrically shielded and soundproofed room with dim lights while the participants had their eyes closed. The participants were instructed to relax and avoid any body movements and drowsiness. EEG activity was recorded from 64 Ag/AgCl electrodes based on the modified international 10‐20 system in conjunction with vertical and horizontal electrooculograms and a mastoid reference electrode; the ground channel was located between the FP_z_ and F_z_ electrodes. The EEG channels were acquired and amplified at a sampling rate of 1000 Hz (Scan 4.5, NeuroScan, Compumedics; El Paso, TX, USA) using a 0.1‐ to 100‐Hz online bandpass filter. Electrode impedances were kept below 5 kΩ, and artifacts due to eye blinks and movements during EEG recording were eliminated by visual inspection and the automatic NG Deluxe 2.6.1 system (NG Deluxe 2.6.1, Applied Neuroscience; St. Petersburg, FL, USA).

##### EEG data preprocessing

The coherence analysis methods used in the present study have been described in previous studies conducted by our research group.^60^ All EEG data were analyzed with the NG Deluxe 2.6.1 system. For the coherence analysis, 19 of the 64 channels with the linked ear reference were selected: FP1, FP2, F7, F3, F_z_, F4, F8, T3, C3, C_z_, C4, T4, T5, P3, P_z_, P4, T6, O1, and O2. The mean, variance, standard deviation, sum of squares, and squared sum of the real (cosine) and imaginary (sine) coefficients of the cross‐spectral matrix were computed across the sliding average of the edited EEG data for all 19 leads, which resulted in a total of 81 and 1539 log‐transformed elements for each participant. The EEG values of each participant at each electrode were computed for each of the following frequency bands: delta (1‐4 Hz), theta (4‐8 Hz), alpha (8‐12 Hz), beta (12‐25 Hz), high beta (25‐30 Hz), and gamma (30‐40 Hz). Next, the following equation was used to determine coherence:

$$\text{Coherence}\;\left(f\right)=\frac{{\left(\sum_{N}\left(a\left(x\right)u\left(y\right)+b\left(x\right)v\left(y\right)\right)\right)}^{2}+{\left(\sum_{N}\left(a\left(x\right)v\left(y\right)-b\left(x\right)u\left(y\right)\right)\right)}^{2}}{\sum_{N}\left(a{\left(x\right)}^{2}+b{\left(x\right)}^{2}\right)\sum_{N}\left(u{\left(y\right)}^{2}+v{\left(y\right)}^{2}\right)},$$

where

$$\begin{array}{l} a\left(x\right)=\text{cosine coefficient for the frequency}\;\left(f\right)\;\text{for channel}\;x,\\ b\left(x\right)=\text{sine coefficient for the frequency}\;\left(f\right)\;\text{for channel}\;x,\\ u\left(y\right)=\text{cosine coefficient for the frequency}\;\left(f\right)\;\text{for channel}\;y,\text{and}\\ v\left(x\right)=\text{sine coefficient for the frequency}\;\left(f\right)\;\text{for channel}\;y. \end{array}$$

##### EEG network calculation

**Figure 1:**
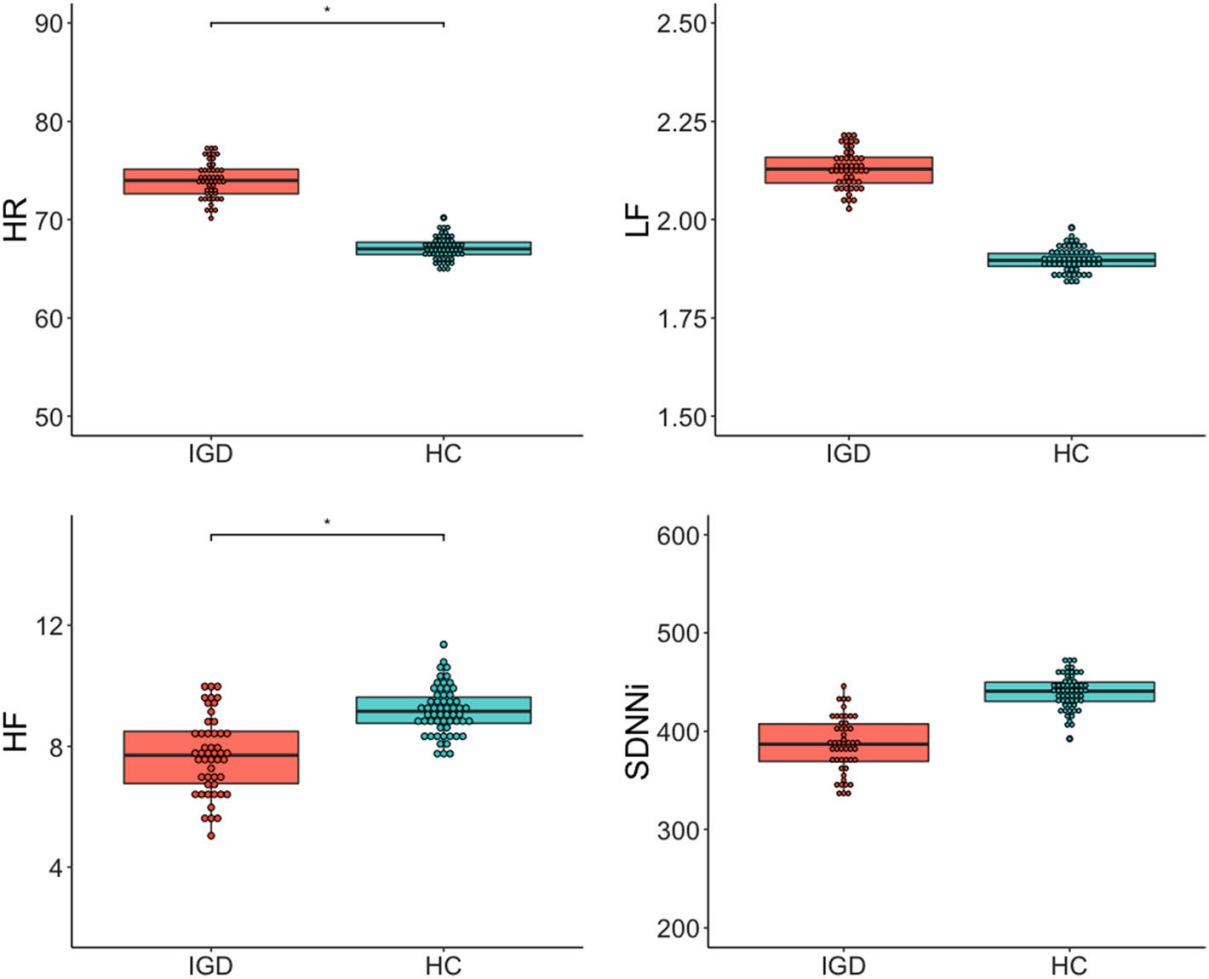
Group comparisons of HR and HRV. *P < .5. The dots represent the predicted values, adjusting for the effect of IQ. HC, healthy control; HF, high frequency; HR, heart rate; HRV, heart rate variability; IGD, Internet gaming disorder; IQ, intelligence quotient; LF, low frequency; SDNNi, the average of the standard deviation of NN interval for each segment of 50 seconds length

The weighted and undirected networks were built based on a coherence adjunct matrix at each frequency band (Figure 1); the vertices of the networks are the selected 19 channels, and the edges are weighted by the coherence value within each pair of vertices. The graph analysis was performed with the Brain Connectivity Toolbox (BCT; http://www.brain‐connectivity‐toolbox.net/) using MATLAB, and the following two parameters were calculated:

1. Characteristic path length (CPL; *L* ^*W*^): the average shortest path length in the network (a shorter [smaller] CPL indicates network integration), which was calculated with BCT using the following equation:

$$L^{w}=\frac{1}{n}\sum i\in N\frac{\sum j\in N,j\neq {\mathrm{id}}_{\mathrm{ij}}^{w}}{n-1}.$$

- Clustering coefficient (CC; *C* ^*W*^): the probability that the nodes neighboring a particular node are connected to each other (this is indicative of the segregation of a network and the average CC was applied for overall network efficacy), which was calculated with BCT using the following equation:

$$C^{w}=\frac{1}{n}\sum i\in N\frac{2t_{i}^{w}}{k_{i}\left(k_{i}-1\right)}.$$

When the CC is large and there is a short CPL, the network is considered to be a small‐world network.

### 2.3 Statistical analysis

Prior to conducting the statistical analyses, outliers in the HRV and EEG parameters were detected and removed (R::Package “car”::outlierTest). Group differences were assessed using a linear regression model or a generalized linear regression model (GLM) after inspection of the data distribution via both visual inspection of the histogram and the Shapiro‐Wilk test. The HF, LF, and all functional connectivity parameters for the EEG network were not normally distributed. These skewed data were analyzed with a gamma GLM. Prior to inputting covariates into the model for group differences, the condition index for multicollinearity was tested (R::Package “perturb”). In addition, the partial eta squared (*η*_*p*_^2^) effect size was calculated for the group comparison (R::Package “lmsupport”).

To assess relationships among the variables that were not normally distributed, Spearman correlation analyses were conducted, and the false discovery rate (FDR) was used for multiple corrections. The plotting of associations between the variables was based on the Spearman correlations with FDR and conducted using the R::Package “qgraph.” All statistical analyses and graphical representations were performed with R version 3.4.4 (R Development Core Team, Vienna, Austria).

## 3 RESULTS

### 3.1 Group differences

#### 3.1.1 Demographic and psychological data

The IGD and HC groups significantly differed in years of education and IQ (*t* = −5.34, *P* < .001, *η*_*p*_^*2*^ = 0.21 and *t* = −4.68, *P* < .001, *η*_*p*_^*2*^ = 0.17, respectively). The IGD group had higher levels of depression, anxiety, impulsivity, and aggressiveness compared with the HC group, even after adjusting for IQ (all *P*s and adj *P*s < .001). Additionally, the IGD group had higher scores on the BIS compared with the HC group, whereas the scores on the BAS did not differ (*P* < .001 and .175, respectively). The AUDIT score also did not differ between the groups (.964). Table 1 presents the detailed data of group differences in demographic and psychological variables.

Only IQ was used as a covariate in further analyses because multicollinearity was high when group, IQ, education, and psychological variables in which group differences emerged were all included in the regression model (condition index greater than 30).^61^

#### 3.1.2 HR and HRV parameters

Figure 1 shows the group comparisons for the HR and HRV parameters. The IGD group had a significantly higher HR than the HC group (*t* = 2.39, *P* = .019, *η*_*p*_^*2*^ = 0.06). In terms of HRV, the GLM analysis revealed that the HF of the IGD group was lower than that of the HC group (*t* = −2.50, *P* = .014, *η*_*p*_^*2*^ = 0.17); LF did not differ between the two groups (*P* > .05). There were no significant group differences in the time domain parameters except that the SDNNi of the IGD group was decreased compared with the that in the HC group when the effect of IQ was not considered (*t* = −2.57, *P* = .012, *η*_*p*_^*2*^ = 0.06); therefore, this variable was selected for further analysis. The supplementary material provides the descriptive statistics of the HR and HRV parameters in detail (Table S1).

#### 3.1.3 EEG functional neural connectivity

**Figure 2:**
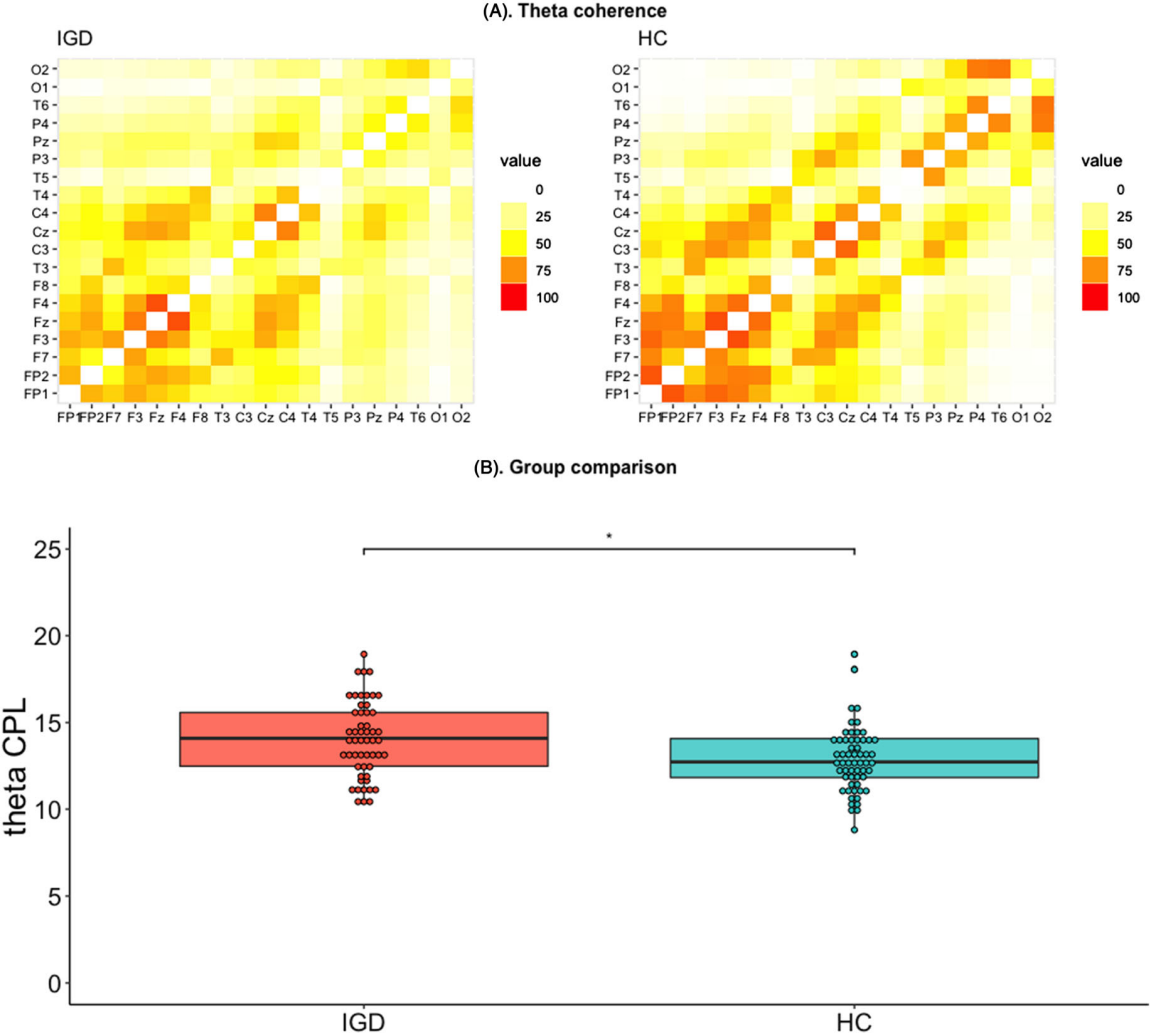
Group comparisons of theta CPL. *P < .05. The upper line represents the theta coherence adjunct matrices of the IGD and HC groups (A). The lower line represents the group comparison of theta CPL (B). The dots represent the predicted values after adjusting for the effect of IQ. CPL was calculated based on theta coherence recorded by EEG. CPL, characteristic path length; EEG, electroencephalography; HC, healthy control; IGD, Internet gaming disorder; IQ, intelligence quotient

For the EEG functional network, the theta CPL was significantly higher in the IGD group than in the HC group (*t* = 2.12, *P* = .037, *η*_*p*_^*2*^ = 0.12; Figure 2); the other frequency band CPL values did not differ between the groups. There were no group differences in CC; therefore, further analyses with CC were not conducted. The supplementary material provides the descriptive statistics of the EEG parameters in detail (Table S2).

### 3.2 Relationships between HRV and EEG functional neural connectivity

In the IGD group, there were significant negative correlations between the SDNNi and slow band CPLs (CPL‐SDNNi: delta, *r* = −0.38, *P*_FDR_ = .0366; theta, *r* = −0.37, *P*_FDR_ = .0464). Contrary to the above results, there was no significant association between HRV‐EEG and CPL in the HC group (all *P*_FDR_s > .050).

## 4 DISCUSSION

The present study aimed to clarify the relationship between ANS activity and brain activity in IGD patients based on neurovisceral integration and verified the study hypotheses, as follows: (a) IGD patients showed elevated HR and decreased HRV (as represented by the HF), and (b) IGD patients exhibited inefficient resting‐state functional neural networks compared with HCs. Subsequently, the third hypothesis was also verified, where (c) there were distinct HRV‐EEG functional connectivity interactions in the IGD patients compared with the HCs. In the IGD group, SDNNi was negatively correlated with slow wave (theta and delta) CPL values. However, the inverse correlations between HRV‐functional connectivity were not significant in the HC group. To the best of our knowledge, the identification of a distinctive neurovisceral correlate in IGD patients is a novel finding in the field of IGD research.

The higher HR and decreased HF‐HRV observed in the IGD patients during a resting state in the present study suggests that a disruption in the cardiac autonomic balance of sympathetic activity and a suppression of the parasympathetic activity underpinning vagal tone. Higher resting HRV is known to be associated with effective functioning of the prefrontal‐subcortical inhibitory circuits, which supports flexible and adaptive responses to environmental demands.^6^, ^62^ Thus, lower HF in patients with IGD than controls in this study may be an index of dysfunctional prefrontal‐subcortical inhibition circuits within CAN, reflecting lower regulatory function among those with IGD. Maladaptive prefrontal‐subcortical inhibition has been reported to be associated with cognitive processing, which plays a critical role in emotional regulation and is found in a wide range of psychopathologies, including depression, anxiety, and schizophrenia.^63^ In particular, the dysfunctional frontal‐subcortical/limbic circuits within the limbic ACC, VMPFC, amygdala, and striatal regions in those with addictive behaviors may be associated with deterioration of stress regulation, craving, and reduced inhibitory control.^64^ Taken together, these findings suggest that decreased HRV in patients with IGD shares features of a maladaptive neurobiological mechanism with other addiction‐related and psychiatric disorders in that it reflects frontal‐subcortical disintegration.

The heightened HR in patients with IGD in this study could represent high levels of autonomic arousal observed in SUD patients, as well as in patients with behavioral addictions such as pathological gambling.^65^ Consistent with this perspective, an elevated HR seemed to be associated with an addictive state or the urge to engage in Internet gaming in the present study, because HR was positively correlated with Y‐IAT score in all groups. Among time domain parameters, only the SDNNi decreased in IGD patients compared with the healthy participants with unadjusted IQ data (*P* < .05). In this study, the length of i in the SDNNi was 50 seconds, so a short‐term variable parasympathetic effect was included when sympathetic tone was detected in the analysis.^59^ In contrast, a simple SDNN can have both parasympathetic and sympathetic tones. In summary, the results of HF‐HRV and SDNNi change in the unadjusted result suggest that patients with IGD may exhibit parasympathetic system abnormalities.

The present finding that an increased theta CPL was indicative of decreased network efficacy in IGD patients after adjusting for IQ supports the findings of numerous previous studies that investigated disrupted functional connectivity in this population.^66^ Theta connectivity can be associated with the regulation of cognitive control and/or integration of sensory information in executive control.^67^, ^68^ Contrary to the present results, theta connectivity has been shown to increase in patients with AUD, depending on psychological conditions, including the severity of depression and anxiety, and in patients with generalized anxiety disorder.^69^, ^70^ In the present study, an increase in theta coherence CPL, which is indicative of decreased integration of theta connectivity, was found. However, it is difficult to conclude that decreased theta connectivity might be a candidate feature IGD that distinguishes patients with this characteristic from patients with AUD, as the depressive and anxiety symptoms were not controlled in this study because of a multicollinearity issue. Thus, it is not possible to compare the findings of this study directly with the results of previous studies on AUD. The number of comparative studies with IGD as a behavioral addiction and AUD as a SUD is insufficient.^71^ Future research to determine whether the decreased theta CPL identified in this study reflects IGD, characteristics commonly found with other addictions, or psychological symptoms is necessary.

The primary finding in the present study was that resting‐state slow wave CPL, including in the theta and delta bands, was negatively correlated with the SDNNi during simultaneous recordings of HRV and EEG in IGD patients. In contrast to the IGD group, there were no resting‐state HRV‐EEG network correlations in the HC group. Thus, it appears that the brain‐body integration required for homeostatic balance was inadequate in IGD patients in the present study compared with healthy individuals, even at rest. According to the neurovisceral integration perspective, HRV itself acts as index of cortical‐subcortical pathways, including connections between the amygdala and medial prefrontal cortex,^72^ and resting‐state functional connectivity of the MPFC is positively correlated with HRV.^73^ Persistent cravings, changes in self‐regulation, cognitive control, and reward circuity within the mesolimbic dopamine system may be related to changes in the brain‐body interaction loop.^12^, ^74^ This study detected a positive association of dysfunctional changes in brain function, accompanied by decreased HRV, with IGD. This relationship may reflect neurovisceral integration. However, as the functional neural connectivity addressed in this study was on a large scale and was measured at the scalp, the present results are not sufficient to directly explain the relationship between HRV and specific brain areas, such as the medial prefrontal cortex‐amygdala connection involved in CAN, and their relation to actual neurovisceral integration. Nevertheless, this is the first study to try to clarify the correlation between brain function and HRV in the addiction field and in IGD. It is necessary to study addictive behavior and brain‐body integration more precisely based on a region‐by‐region brain approach, and this study may provide the foundation for future research.

**Figure 3:**
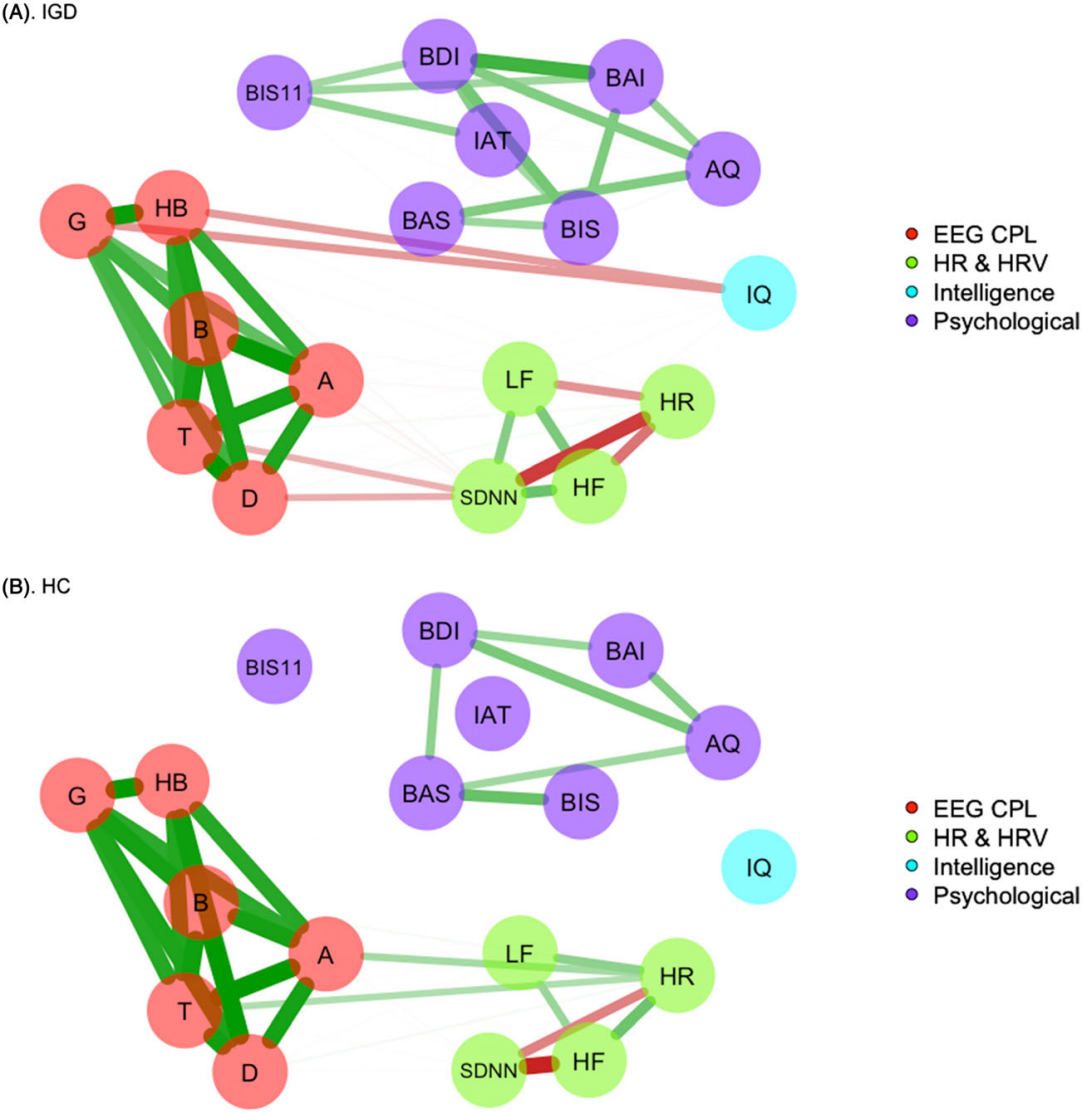
Spearman correlational plot of the variables for the IGD and HC groups. Edges are represented by the weight (Spearman correlation coefficients) and sign of association between the nodes (variables): (1) Weight refers to the intensity of association and is represented by the thickness of the lines and (2) sign refers to positive (green line) and negative (red line) relationships. Only edges that were significant after the FDR correction are represented. The upper line (A) represents the correlations in the IGD group, and the lower line (B) represents the correlations in the HC group. A, alpha; AQ, Buss‐Perry Aggression Questionnaire; B, beta; BAI, Beck anxiety inventory; BDI2, Beck depression Inventory‐2; BIS11, Barrett impulsiveness Scale‐11; BIS/BAS, the Behavioral Inhibition System/Behavioral Approach System (BIS/BAS) scales; D, delta; EEG CPL, characteristic path length calculated from electroencephalographic coherence at each band below; FDR, false discovery rate; G, gamma; HB, high‐beta; HC, healthy control; HF, high frequency; HR, heart rate; HRV, heart rate variability; IAT, Young Internet addiction test; IGD, Internet gaming disorder; IQ, intelligence quotient; LF, low frequency, SDNNi, the average of the standard deviation of NN interval for each segment of 50 seconds length; T, theta

In addition to verifying the main hypothesis, we explored the relationships among variables, including HRV, the EEG functional neuronal network, psychological variables, and global intellectual functioning. Figure 3 illustrates the patterns of relationships among the variables in the IGD and HC groups. The edges represent the weight and sign of the association between the nodes, as follows: (a) Weight refers to the intensity of the association and is represented by the thickness of the lines, and (b) the sign refers to positive (green line) or negative (red line) relationships. In the IGD group, the Y‐IAT score was significantly correlated with scores on the BDI2, BIS11, and BIS (all *P*_FDR_ < .05). Additionally, the fast band, including high beta and gamma CPLs, was negatively correlated with IQ (all *P*_FDR_ < .05). No significant direct relationships were observed between HR/HRV and EEG parameters or self‐reported psychological variables after the FDR correction for multiple comparisons. In the HC group, the correlations among psychological variables were sparser compared with those in the IGD group. In the entire sample, the Y‐IAT score was positively correlated with scores on the BDI2, BIS11, AQ, BAI, and BIS (*r* = 0.72, *P* < .001; *r* = 0.61, *P* < .001; *r* = 0.60, *P* < .001; *r* = 0.56, *P* < .001; and *r* = 0.52, *P* < .001, respectively). The Y‐IAT score tended to be negatively related to the SDNNi, but this result did not meet hold following the correction for multiple comparisons (*r* = −0.20, 0.044, *P*_FDR_ > .05). The detailed results of the correlations after FDR corrections are presented in the supplementary data (Table S3 for the IGD and HC groups and Table S4 for the entire sample).

It is possible that predispositional factors might account for the atypical ANS and ANS‐brain correlations observed in the IGD patients in the present study. The IGD group had a high BIS score, which, according to Gray's theory, is related to low parasympathetic and vagal tone, influences depression and anxiety levels, and induces other inhibitory behavioral manifestations.^34^ In the present study, there was no direct association between BIS score and the physiological markers, but the psychological symptoms were associated with increased HR. A previous study from our research group showed that psychological symptoms were mediators between BIS score and the degree of Internet addiction.^30^ Another predispositional factor associated with brain‐body correlations is general intelligence level, as indexed by IQ. In the present study, the IGD group had a lower education level and a lower IQ than the HC group and IQ was associated with HR, the SDNNi, and the fast wave (high beta and gamma) functional networks. However, HRV, as represented by HF, remained lower in the IGD group after controlling for IQ. IQ was also associated with the severity of depression in the present study. Therefore, it is not plausible that the degradation of HRV and network efficiency in the IGD group was solely due to predispositional cognitive abilities. Taken together, these findings suggest that brain‐body integrative activity could influence broad aspects of IGD, including addictive, behavioral, emotional, and cognitive factors.

Decreased HRV and maladaptive ANS–central nervous system (CNS) interactions can result in a vicious cycle of worsening regulation of emotional, behavioral, and cognitive factors in IGD patients. Subsequently, this phenomenon might enhance the vulnerability of IGD patients to stress and lead to isolation from daily life activities such as school, work, and interpersonal relationships. From a long‐term perspective, this may result in a general deterioration of medical health, including decreased immune system function and increased cardiovascular risks, as observed in patients with SUD.^15^ Moreover, HRV may represent a prognostic state or have predictive value with respect to the treatment response per se.^75^ The present findings suggest that there are clinical implications that will require the careful observation of the prognoses of IGD patients from a comprehensive and long‐term perspective. In addition to traditional interventions such as medication, behavior modification, and motivation enforcement methods, therapeutic approaches that effectively “reprogram” the addicted brain‐body circuit may be useful for treatment and relapse prevention of IGD. Mindfulness‐based interventions, neurofeedback, and biofeedback methods might be effective for retraining the neurovisceral interaction pathway.

The present findings will contribute to a comprehensive understanding of the neurobiological markers of IGD in terms of traits, psychological symptoms, cognition, ANS activity, and brain functional state. Because HRV and EEG parameters may fluctuate based on the time of measurement, the present study had methodological advantages in that the results will validate real‐time neurovisceral interactions because of the fact that EEG and HRV were measured simultaneously. Additionally, because the participants were not taking medication, it was possible to control the exogenous effects of drugs that might have crucial consequences on psychophysiological signs. Furthermore, there was no significant difference between the IGD and HC groups on the AUDIT, which assess the tendency for alcohol addiction (*P* > .05).

The present study also had several limitations that should be considered. First, the majority of IGD patients were male (50 males vs three females) and, therefore, the effects of gender on HRV and the functional networks could not be closely examined. Second, respiration rate, which might influence the respiratory sinus arrhythmia associated with HRV, was not assessed during ECG recording. Third, this study used a cross‐sectional design, and, as a result, a causal relationship could not be inferred and long‐term predictions could not be made. Fourth, the severity of Internet addiction and psychological variables were measured by a self‐reported instrument. Fifth, only IGD patients and HCs were included in this study. In addition, this study did not control for the influence of psychological variables, such as depression, anxiety, and impulsivity in the analysis. This study was not conducted using psychological variables as covariates/extraneous variables for following reasons: (a) Doing so increased the multicollinearity of the models, and (b) psychological phenomena, including depression, anxiety, and impulsiveness, can be linked to and interact with Internet addiction. Hence, it may be difficult to identify the effects of Internet addiction per se when excluding psychological symptoms. Thus, it remains unclear whether the physiological characteristics of the IGD patients observed in the present study reflect the characteristics of IGD, the characteristics of addiction, or, more broadly, the general characteristics of psychiatric disorders. Future large‐scale long‐term studies comparing IGD with other addictions and other psychiatric disorders, including depressive disorder or externalizing disorder, without addictive behaviors will be needed to clarify these issues.

## 5 CONCLUSIONS

In the present study, the IGD patients were distinguishable from HCs based on distinctive features that included an elevated HR, decreased HRV (as indexed by the HF), and decreased theta functional connectivity (as measured by graph theory using EEG parameters). Furthermore, the IGD patients exhibited correlations in HRV‐functional neural connectivity, whereas the HCs did not. The present findings will contribute to a more comprehensive understanding of the maladaptive neurovisceral integration features associated with IGD.

## DISCLOSURE/CONFLICT OF INTEREST

The authors declare no conflict of interest.

## AUTHORS CONTRIBUTIONS

SMP contributed to study conception and design, analyzing data, interpretation of the findings, and writing the manuscript. JYL, ARC, BMK, JSJ, and MKP contributed to data collection and assisted with EEG data analysis. IYK, JP, JC, and SJH contributed to HRV data analysis. J‐SC contributed to study conception and design, interpretation of the findings, critical revision of the manuscript, and supervision. All authors critically reviewed content and approved the final version for publication.

## Supporting information


**Table S1.** Group differences in HR and HRV
**Table S2**. Group differences in the EEG functional neural network
**Table S3**. Spearman correlational coefficients among variables in the IGD and HC groups
**Table S4**. Spearman correlational coefficients among variables in the entire sample
