# Supplementary material for: Maladaptive neurovisceral interactions in patients with Internet gaming disorder: A study of heart rate variability and functional neural connectivity using the graph theory approach
Source: Addict Biol. 2019 Jul 12;25(4):e12805. doi: 10.1111/adb.12805 (PMC7317587; doi:10.1111/adb.12805)
Supplement: Supplementary file 1 — Table S1. Group differences in HR and HRV Table S2. Group differences in the EEG functional neural network Table S3. Spearman correlational coefficients among variables in the IGD and HC groups Table S4. Spearman correlational coefficients among variables in the entire sample [file ADB-25-e12805-s001.pdf]

Table S1. Group differences in HR and HRV

	IGD (n=50)		HC (n=54)				
	Mean	SE	Mean	SE	*t*	*P*	η_p^2
HR	74.42	1.56	67.07	1.39	2.39	0.019	0.06
LF	453.26	61.89	527.13	66.47	0.46	0.646	-
HF	126.44	12.66	109.42	7.08	-2.50	0.014	0.17
SDNN	41.38	3.05	42.74	2.52	-0.18	0.854	-
SDNNi	385.72	14.82	439.98	14.95	-1.43	0.157	-
RMSSD	43.53	4.39	44.77	3.25	-0.28	0.778	-
pNN50	69.41	2.36	73.30	1.85	-0.36	0.723	-

The effect of IQ was adjusted. IGD = Internet gaming disorder, HC = healthy control. IQ = intelligence quotient. HR = hear rate. HRV = heart rate variability; LF = low frequency, HF = high frequency, SDNN = standard deviation of NN Interval. SDNNi **=** standard deviation of NN interval index, RMSSD = root mean square of difference of the NN intervals, pNN50 = percentage of the NN intervals that differ by more than 50 milliseconds.

Table S2. Group differences in the EEG functional neural network

	IGD (n=50)		HC (n=54)				
	Mean	SE	Mean	SE	*t*	*P*	η_p^2
CPL							
Delta	7.99	0.54	8.77	0.68	1.96	0.053	
Theta	7.34	0.5	8.11	0.62	2.12	0.037	0.12
Alpha	6.95	0.52	7.79	0.63	1.98	0.050	
Beta	7.88	0.55	8.40	0.63	1.90	0.061	
Highbeta	7.80	0.59	7.69	0.61	1.44	0.153	
Gamma	7.38	0.63	7.05	0.57	1.24	0.219	
CC							
Delta	0.13	0.01	0.13	0.01	-1.04	0.300	
Theta	0.13	0.02	0.13	0.01	-1.46	0.147	
Alpha	0.14	0.02	0.13	0.01	-1.39	0.169	
Beta	0.13	0.01	0.13	0.01	-1.31	0.193	
Highbeta	0.17	0.02	0.18	0.02	-0.81	0.418	
Gamma	0.13	0.01	0.21	0.02	-0.82	0.414	

The effect of IQ was adjusted. IGD = Internet gaming disorder, HC = healthy control. IQ = intelligence quotient. EEG = electroencephalographic, CPL = characteristic path length, CC = clustering coefficients.

Table S3. Spearman's correlational coefficients among variables in the IGD and HC groups

	Self-reported measure							IQ	HR	HRV			EEG CPL					
IGD / HC	IAT	BDI2	BAI	AQ	BIS11	BIS	BAS	IQ	HR	LF	HF	SDNNi	Delta	Theta	Alpha	Beta	Highbeta	Gamma
IAT	1	0.46**	0.33	0.3	0.44**	0.37*	0.17	0.16	0.27	-0.18	-0.08	-0.07	-0.12	-0.15	-0.19	-0.15	-0.09	-0.03
BDI2	0.28	1	0.73***	0.5**	0.37*	0.55***	0.18	-0.13	0.16	-0.04	-0.23	-0.02	-0.07	-0.08	-0.1	-0.11	-0.14	-0.16
BAI	0.16	0.47**	1	0.44**	0.39*	0.52***	0.3	-0.26	0.01	0.13	-0.09	-0.09	0.04	0.05	0.03	0	-0.04	-0.08
AQ	0.28	0.21	0.42**	1	0.18	0.16	0.51**	-0.07	0.08	-0.01	0.07	0.04	0.02	0.04	0.01	-0.02	-0.01	-0.05
BIS11	0.13	0.07	0.21	0.16	1	0.23	0.04	0.05	0.07	-0.11	-0.18	-0.07	-0.12	-0.1	-0.07	-0.12	-0.23	-0.31
BIS	0.26	0.26	0.41**	0.52***	0.2	1	0.41*	0.09	-0.09	0.12	-0.04	0.11	0.04	0.05	0.05	0.04	-0.03	-0.08
BAS	0.08	0.11	0.29	0.35	0.29	0.51***	1	-0.12	0	0.2	0.1	0.08	0.04	0.03	0.04	0.04	0.02	0.01
IQ	0.28	-0.23	0.18	0.16	0.04	0.07	-0.01	1	-0.15	-0.1	0.24	0.34	-0.33	-0.32	-0.26	-0.32	-0.39*	-0.4*
HR	-0.09	-0.13	-0.1	0.03	-0.16	0.15	0.17	-0.14	1	-0.41*	-0.53***	-0.8***	0.18	0.14	0.05	0.09	0.09	0.1
LF	0.21	0.1	-0.04	-0.17	0.14	0.05	-0.09	0.17	-0.44**	1	0.51**	0.48**	0.05	0.09	0.18	0.14	0.03	-0.01
HF	0.12	0.09	-0.03	-0.1	0.11	-0.08	-0.07	0.19	-0.19	0.23	1	0.59***	-0.06	-0.05	0.03	-0.03	-0.03	-0.06
SDNNi	0.01	0.16	0.11	-0.07	0.23	-0.14	-0.05	0.1	-0.82***	0.53***	0.31	1	-0.38*	-0.37*	-0.31	-0.35	-0.33	-0.3
Delta	0.09	0.12	-0.23	-0.12	-0.06	-0.02	-0.17	-0.16	-0.18	0.3	0.17	-0.02	1	0.97***	0.91***	0.93***	0.86***	0.76***
Theta	0.1	0.09	-0.29	-0.19	-0.1	-0.02	-0.18	-0.2	-0.2	0.33	0.17	0.01	0.97***	1	0.95***	0.96***	0.84***	0.71***
Alpha	0.11	0.12	-0.27	-0.24	-0.08	0.01	-0.14	-0.23	-0.16	0.34	0.19	0	0.92***	0.97***	1	0.97***	0.78***	0.6***
Beta	0.01	0.11	-0.3	-0.2	-0.07	-0.03	-0.13	-0.28	-0.11	0.22	0.22	-0.04	0.93***	0.96***	0.95***	1	0.89***	0.75***
Highbeta	-0.08	0.12	-0.24	-0.14	-0.01	-0.07	-0.12	-0.27	-0.13	0.16	0.14	-0.03	0.87***	0.86***	0.84***	0.94***	1	0.95***
Gamma	-0.1	0.11	-0.24	-0.13	-0.03	-0.11	-0.17	-0.23	-0.14	0.16	0.13	-0.02	0.85***	0.83***	0.79***	0.9***	0.98***	1

* = P_{FDR} < 0.05, ** = P_{FDR} < 0.01, *** = P_{FDR} < 0.001. The upper right section represent the results in the IGD group and the down left section represent the results in the HC group. IGD = Internet gaming disorder, HC = healthy control. IAT = Korean version of the Young's Internet Addiction Test, BDI2 = Korean version of the Beck Depression Inventory-2, BAI = Korean version of the Beck Anxiety Inventory, AQ = Korean version of the Buss–Perry Aggression Questionnaire, BIS11 = Korean version of the Barrett Impulsiveness Scale-11, BIS/BAS = Korean version of the Behavioral Inhibition System/Behavioral Approach System (BIS/ BAS) scales. IQ = intelligence quotient. HR = hear rate. HRV = heart rate variability; LF = low frequency, HF = high frequency, SDNN = standard deviation of NN Interval. SDNNi = standard deviation of NN interval index, RMSSD = root mean square of difference of the NN intervals, pNN50 = percentage of the NN intervals that differ by more than 50 milliseconds. EEG electroencephalography, CPL = characteristic path length. FDR = false discovery rate.

Table S4. Spearman's correlational coefficients among variables in the entire sample

	Self-reported measure							IQ	HR	HRV			EEG CPL					
	IAT	BDI-2	BAI	AQ	BIS11	BIS	BAS	IQ	HR	LF	HF	SDNN	Delta	Theta	Alpha	Beta	Highbeta	Gamma
IAT	1	0.72***	0.56***	0.6***	0.61***	0.52***	0.21	-0.23	0.34**	-0.05	0.09	-0.2	-0.09	-0.11	-0.12	-0.1	-0.04	0
BDI2		1	0.79***	0.64***	0.54***	0.59***	0.22	-0.37***	0.27*	-0.03	-0.05	-0.14	-0.06	-0.08	-0.08	-0.07	-0.04	-0.04
BAI			1	0.59***	0.52***	0.58***	0.31**	-0.34**	0.16	0.04	-0.01	-0.14	-0.08	-0.09	-0.09	-0.09	-0.07	-0.07
AQ				1	0.42***	0.47***	0.45***	-0.24	0.28*	-0.09	0.08	-0.16	-0.09	-0.11	-0.15	-0.12	-0.05	-0.04
BIS11					1	0.4***	0.21	-0.19	0.15	-0.02	-0.01	-0.07	-0.12	-0.13	-0.11	-0.11	-0.1	-0.14
BIS						1	0.47***	-0.1	0.16	0.06	0	-0.1	-0.03	-0.03	-0.02	-0.02	-0.04	-0.07
BAS							1	-0.14	0.15	0.04	0.06	-0.03	-0.1	-0.11	-0.08	-0.07	-0.05	-0.07
IQ								1	-0.26*	0.05	0.13	0.3**	-0.17	-0.18	-0.17	-0.23	-0.29**	-0.31**
HR									1	-0.42***	-0.33**	-0.82***	-0.04	-0.08	-0.09	-0.03	-0.01	0
LF										1	0.36**	0.51***	0.21	0.24	0.28*	0.19	0.1	0.07
HF											1	0.41***	0.03	0.03	0.08	0.06	0.04	0.01
SDNN												1	-0.15	-0.12	-0.1	-0.16	-0.17	-0.17
Delta													1	0.97***	0.92***	0.93***	0.86***	0.8***
Theta														1	0.97***	0.96***	0.84***	0.76***
Alpha															1	0.96***	0.81***	0.69***
Beta																1	0.92***	0.82***
Highbeta																	1	0.96***
Gamma																		1

* = $P_{FDR} < 0.05$, ** = $P_{FDR} < 0.01$, *** = $P_{FDR} < 0.001$. IAT = Korean version of the Young's Internet Addiction Test, BDI-2 = Korean version of the Beck Depression Inventory-2, BAI = Korean version of the Beck Anxiety Inventory, AQ = Korean version of the Buss–Perry Aggression Questionnaire, BIS11 = Korean version of the Barrett Impulsiveness Scale-11, BIS/BAS = Korean version of the Behavioral Inhibition System/Behavioral Approach System (BIS/ BAS) scales. IQ = intelligence quotient. HR = hear rate. HRV = heart rate variability; LF = low frequency, HF = high frequency, SDNN = standard deviation of NN Interval. SDNNi = standard deviation of NN interval index, RMSSD = root mean square of difference of the NN intervals, pNN50 =

percentage of the NN intervals that differ by more than 50 milliseconds. EEG electroencephalography, CPL = characteristic path length. FDR = false discovery rate.
